# Hypoparathyroidism, deafness and renal dysplasia syndrome caused by a *GATA3* splice site mutation leading to the activation of a cryptic splice site

**DOI:** 10.3389/fendo.2023.1207425

**Published:** 2023-08-04

**Authors:** Catarina I. Gonçalves, Josianne N. Carriço, Omneya M. Omar, Ebtesam Abdalla, Manuel C. Lemos

**Affiliations:** ^1^ CICS-UBI, Health Sciences Research Centre, University of Beira Interior, Covilhã, Portugal; ^2^ Department of Pediatrics, Faculty of Medicine, Alexandria University, Alexandria, Egypt; ^3^ Department of Human Genetics, Medical Research Institute, Alexandria University, Alexandria, Egypt

**Keywords:** HDR syndrome, hypoparathyroidism, deafness, renal dysplasia, GATA3, splice site mutation, cryptic splice site

## Abstract

The HDR syndrome is a rare autosomal dominant disorder characterised by Hypoparathyroidism, Deafness, and Renal dysplasia, and is caused by inactivating heterozygous germline mutations in the *GATA3* gene. We report an 11-year-old girl with HDR syndrome caused by a heterozygous mutation located at the splice acceptor site of exon 5 of the *GATA3* gene (NM_001002295.2: c.925-1G>T). Functional studies using a minigene assay showed that this splice site mutation abolished the normal splicing of the *GATA3* pre-mRNA and led to the use of a cryptic splice acceptor site, resulting in the loss of the first seven nucleotides (TCTGCAG) of exon 5 in the *GATA3* mRNA. These findings increase the understanding of the mechanisms by which *GATA3* splicing mutations can cause HDR syndrome.

## 1 Introduction

The HDR syndrome (OMIM 146255), also known as Barakat syndrome, is a rare autosomal dominant disorder characterised by Hypoparathyroidism (H), Deafness (D), and Renal dysplasia (R), and is caused by germline mutations of the *GATA3* gene (1, 2).

The primary hypoparathyroidism manifests as low serum concentrations of parathyroid hormone (PTH) leading to symptomatic or asymptomatic hypocalcemia (3). The deafness is usually bilateral, sensorineural, and more evident at higher frequencies (4, 5). The renal abnormalities can manifest as renal aplasia or hypoplasia, vesicoureteral reflux, and renal cysts that may cause compression and deformities leading to renal failure (6).

The clinical expression of each component of the disorder can vary widely (2). Although the hearing loss is commonly diagnosed during childhood, the hypocalcemia and renal abnormalities often stay asymptomatic and undiagnosed for several years, particularly when there is no family history to alert to this diagnosis (7). The type of underlying mutation may influence the severity and age of onset of each HDR feature (2).

The *GATA3* gene is located on chromosome 10p14, comprises six exons and encodes a 444 amino acid protein. The GATA3 protein is a dual zinc-finger transcription factor that is expressed in the developing parathyroid, inner ear, and kidneys (1, 2). In the year 2000, heterozygous loss-of-function mutations of *GATA3* were found to be responsible for the HDR syndrome (8). Since then, mutations in *GATA3* have been reported in at least 124 kindreds, consisting of 40% frameshift deletions or insertions, 23% missense mutations, 14% nonsense mutations, 6% splice site mutations, 1% inframe deletions or insertions, 15% whole-gene deletions, and 1% whole-gene duplications (2).

We present the clinical and genetic characteristics of a patient with HDR syndrome, and the functional characterization of a splice site mutation in the *GATA3* gene.

## 2 Materials and methods

### 2.1 Clinical studies

The patient is an 11-year-old girl, born to non-consanguineous Egyptian parents with unremarkable family histories. Since early life, she suffered several episodes of convulsions and tetany. The first episode occurred at the age of 14 days, during which hypocalcaemia was confirmed. This was attributed to vitamin D deficiency and treated accordingly. Since then, she suffered multiple episodes of convulsions with frequent hospital visits to receive intravenous calcium. At the age of 3 years, an audiogram revealed bilateral severe sensorineural hearing impairment. Upon current admission, physical examination revealed spasm of hands and feet. She had normal facial appearance, no dysmorphic features, and no skeletal abnormalities. She was wearing hearing aids. Systemic examination was unremarkable including cardiac examination. Laboratory assessment revealed low total serum calcium 5.2 mg/dL (ref: 8.8-10.8), low PTH concentration 9.1 pg/mL (ref: 9-52), and high serum phosphorus 10 mg/dL (ref: 4-7). An abdominal ultrasound showed a simple cyst (1.5 x 1.6 cm) with thin wall and clear content in the mid-zone of the left kidney with normal right kidney and normal cortical echogenicity of the kidneys. Magnetic Resonance Imaging (MRI) revealed absent basal ganglia calcification.

### 2.2 Genomic deoxyribonucleic acid sequencing

The genetic studies were approved by the Institutional Ethics Committees of both the Faculty of Health Sciences, University of Beira Interior (Ref: CE-FCS-2013-017) and the Medical Research Institute, University of Alexandria (Ref: IORG0008812). Written informed consent was obtained from the patient’s legal guardian. DNA was extracted from peripheral blood leucocytes of the patient and her unaffected mother (the unaffected father was unavailable for the study) using previously described methods (9). The patient was screened for mutations in *GATA3* by polymerase chain reaction (PCR) amplification of the six coding exons and exon–intron boundaries, and bidirectional sequencing using a CEQ DTCS sequencing kit (Beckman Coulter, Fullerton, CA, USA) and an automated capillary DNA sequencer (GenomeLab TM GeXP, Genetic Analysis System, Beckman Coulter). Primer sequences were designed using Primer3Plus (10) (available upon request). The *GATA3* variant was analyzed by Franklin (Genoox Ltd, https://franklin.genoox.com/) and classified according to American College of Medical Genetics and Genomics (ACMG) criteria (11). The nomenclature of the variant was based on the *GATA3* cDNA reference sequence (GenBank accession number NM_001002295.2).

### 2.3 *In silico* prediction

To predict the effect of the splice site variant, we applied the bioinformatic program NNSplice (http://fruitfly.org/seq_tools/splice.html) that uses machine learning to predict potential splice sites, with a score ranging from 0 (low) to 1 (high) (12).

### 2.4 *In vitro* functional studies

To assess the effect of this splice site variant on the messenger ribonucleic acid (mRNA) of *GATA3*, we used a minigene technique (13). Using the patient genomic DNA as template, the wild-type and mutant alleles of the *GATA3* exon 5, along with 384 base-pairs (bp) of 5’ and 423 bp of 3’ intronic flanking sequences were amplified by PCR using iProof High-Fidelity DNA Polymerase (Bio-Rad Laboratories, Hercules, CA, USA) with the following oligonucleotides: forward 5’-CTGACTGACATATGCTGAAAGCCCAGTTCCAAAA-3’, and reverse 5’-TCAGTCAGAGATCTCCCTGCCACACATTACAATTC-3’. Both oligonucleotides carry NdeI and BglII restriction enzyme sites at the 5’ end, respectively. These restrictions enzymes were used to clone the PCR product into the pcAT7-Glo1 plasmid (a kind gift from Dr. Kristen W. Lynch, Perelman School of Medicine, University of Pennsylvania, USA). COS-7 cells were cultured in a 6-well plate, in Dulbecco’s modified Eagle medium, with 4.5 g/L glucose, L-glutamine, sodium pyruvate, 1.5 g/L NaHCO_3_ (PAN-Biotech GmbH, Aidenbach, Germany), and supplemented with 10% fetal bovine serum and 1% penicillin/streptomycin at 37°C in 5% CO_2_. When a confluence of 60-70% (or 0.3 X 10^6^ cells per well) was achieved, the cultured cells were transfected with 5 µg of the minigene plasmid DNA using Xfect transfection reagent (Takara Bio USA, San Jose, CA, USA), according to the manufacturer’s protocol. Total RNA was harvested from transfected cells, after 24 h, using QIAshredder spin columns (QIAGEN, Hilden, Germany) and RNeasy Mini kit (QIAGEN, Hilden, Germany) following the manufacturer’s recommendations. Reverse transcription reactions were performed with 600 ng of the total extracted RNAs using the RevertAid First Strand cDNA Synthesis kit (Thermo Fisher Scientific Baltics UAB, Vilnius, Lithuania). PCR amplifications were performed with the generated cDNAs as template, using the oligonucleotides: Act 5’-TTCGGCTTCTGGCGTGTGACCGGCGGCTCTAGC-3’ and ActT7R 5’-CACAGTCGAGGCTGATCAGCGG-3’. The amplified products were then sequenced with the CEQ DTCS sequencing kit (Beckman Coulter, Fullerton, CA, USA) and an automated capillary DNA sequencer (GenomeLab TM GeXP, Genetic Analysis System, Beckman Coulter).

## 3 Results

**Figure 1:**
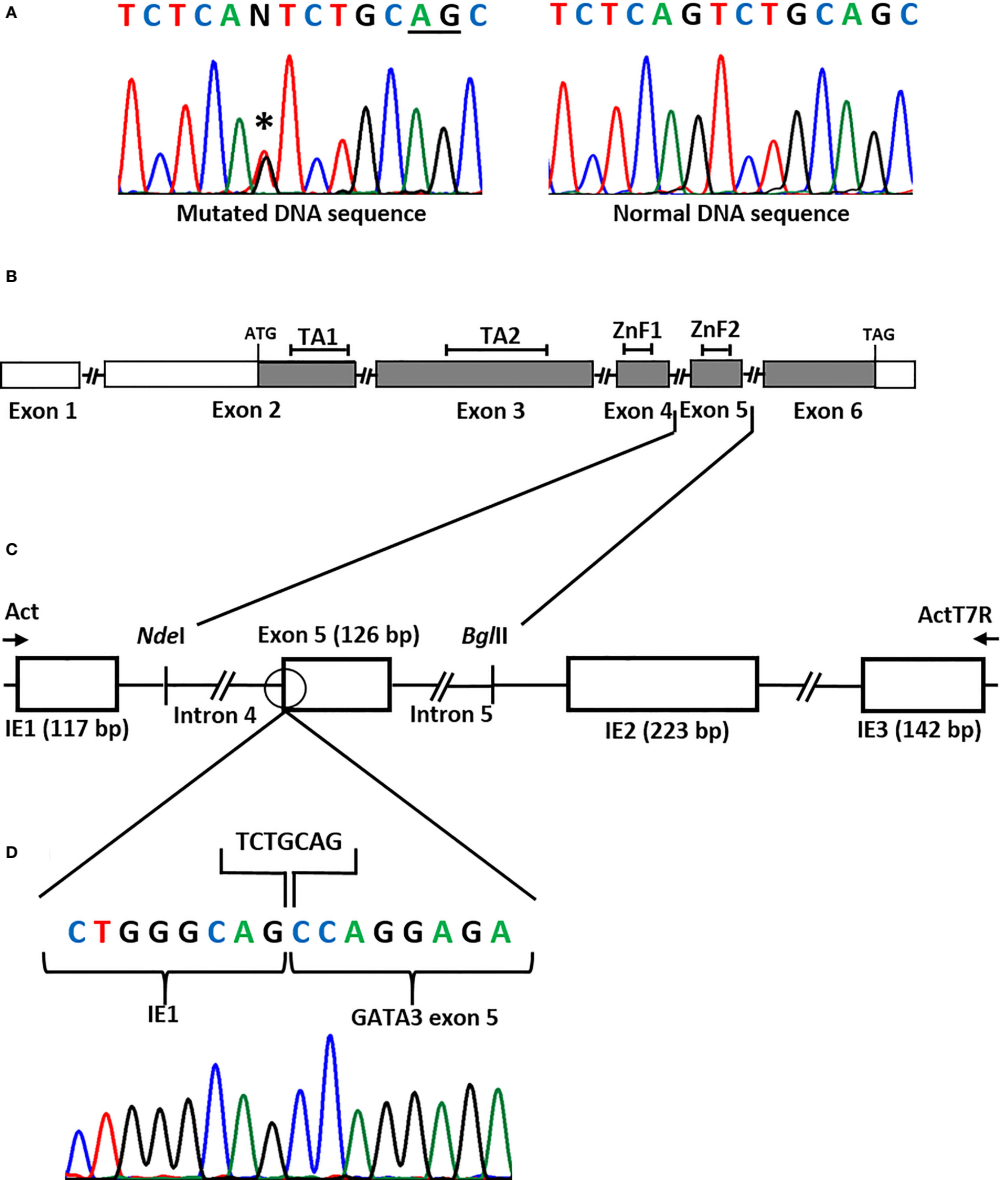
**(A)** DNA sequence of the *GATA3* exon 5 splice acceptor site showing a heterozygous splice site mutation (NM_001002295.2: c.925-1G>T) (asterisk) in the patient. Underlined nucleotides (AG) represent the cryptic splice acceptor site used by the spliceosome. **(B)** Representation of the *GATA3* gene. Boxes represent the exons, filled boxes represent the coding regions, open boxes represent non-coding regions, connecting lines represent the introns (not drawn to scale). TA1, Transactivating domain 1; TA2, Transactivating domain 2; ZnF1, Zinc Finger 1; ZnF2, Zinc Finger 2. The ATG (translation start) codon is in exon 2 and the TAG (stop) codon is in exon 6. **(C)** Minigene assay to assess splicing. The patient had a G>T substitution in the splice acceptor site (open circle) in intron 4 of the *GATA3* gene. To check its effect on the splicing of the transcript, the wild-type and the mutant sequences of exon 5, together with the flanking introns (introns 4 and 5), were cloned into the NdelI and BglII restrictions sites of the pcAT7-Glo1 vector, between intrinsic exons IE1 and IE2. The constructs were transfected into COS-7 cells and RNA was extracted. Reverse Transcription (RT)-PCR using Act and ActT7R flanking primers (arrows) amplified the spliced products. Bp, base-pair. **(D)** DNA sequence of the spliced product showing the loss of seven nucleotides (TCTGCAG) at the beginning of exon 5 due to the use of an alternative splice acceptor site (TCTGCAG).

DNA sequencing of the *GATA3* gene in the patient revealed a heterozygous variant in the splice acceptor site of exon 5 (NM_001002295.2: c.925-1G>T) (**Figure 1A**). The variant was absent in the Genome Aggregation Database (gnomAD) (14). The variant was not found in her unaffected mother. Her unaffected father was unavailable for the study.

The bioinformatic program NNSplice indicated that the normal splice acceptor site of exon 5 had a score of 0.89 and that the next best potential splice site was located seven nucleotides downstream, with a score of 0.93.

The functional studies using the minigene technique showed that the splice site variant abolished the normal splicing of the *GATA3* pre-mRNA and that a cryptic splice acceptor site in exon 5 was used instead. This resulted in the loss of the first seven nucleotides (TCTGCAG) of exon 5 in the *GATA3* mRNA (**Figures 1B–D**).

According to the available evidence, the variant fulfilled the ACMG criteria for “Pathogenic” (criteria PVS1, PS3, PM2).

## 4 Discussion

Our study of an Egyptian girl with HDR syndrome identified a *GATA3* mutation in the splice acceptor site of exon 5 (c.925-1G>T). We demonstrated that the loss of this splice site leads to the use of a cryptic splice acceptor site, located seven nucleotides downstream, that presents a surrounding splice junction sequence similar to the splice junction consensus sequence (N-Y12-14NYAG) (15). This leads to a frameshift that is predicted to produce a missense peptide with a termination at codon 355, resulting in the loss of the GATA3 ZnF2 domain.

The way by which the splicing machinery acts in identifying and removing introns is a central and a conserved step of gene expression in all eukaryotes. RNA splicing depends on the recognition of nucleotide sequences located at the exon-intron boundaries, which include the highly conserved AG and GT dinucleotides at the splice acceptor and donor sites, respectively (16). Mutations that change splicing consensus sequences are often associated with diseases (17, 18). These mutations may result in abnormal splicing through exon skipping, intron retention, or activation of cryptic splice sites (19). Cryptic splice sites have similar splicing consensus sequences, but are not normally used in RNA splicing and are only activated when the authentic splice site is lost as the result of a mutation (20). The consequences of splice site mutations can sometimes be predicted using bioinformatic programs (12), but ultimately, functional studies are needed to confirm what happens at the cellular level. Cell-based analysis of minigene splicing is widely used to investigate the effects of sequence variants on RNA splicing (13). This is usually carried out by cloning the relevant exons of the gene into a plasmid containing endogenously expressed exons and analyzing the transcribed RNA (13).

About 6% of *GATA3* mutations associated with HDR are splice site mutations and it is interesting to note that these are located exclusively in introns 4 and 5 (2). Only four splice site mutations have been studied for their functional consequences. These consist of c.924 + 4_924 + 19del (21) and c.924 + 5G>C (22) that result in skipping of exon 4, and c.1051-1G>T (23) and c.1051-2A>G (24) that result in the use of an alternative splice acceptor site in exon 6.

The mutation found in our patient (c.925-1G>T) was also identified in an Italian patient with hearing loss (25), but no clinical details or functional studies for this patient were presented. Thus, our study is the first to demonstrate the mechanisms by which this mutation disrupts the function of GATA3.

Our patient presented the full triad of the syndrome since early age, which is in agreement with the type of mutation. A review of 177 reported HDR patients showed that the average age of diagnosis of hypoparathyroidism, deafness, and renal defects, was 15.3, 7.5, and 14.0 years, respectively (2). However, GATA3 protein-truncating mutations (frameshift, nonsense, and splice site), which are likely to have a more severe effect than missense mutations, were associated with an earlier expression of the disorder (2). Our patient had no family history of HDR. Therefore, she likely represents a sporadic case caused by a *de novo* mutation, as occurs in approximately half of HDR patients (2).

In conclusion, our results increase the understanding of the mechanisms by which *GATA3* splicing mutations can cause HDR syndrome.

## Data availability statement

The datasets presented in this study can be found in an online repository. The name of the repository and ID number(s) can be found below: LOVD - 0000436803 (phenotype), 0000325519 (DNA seq), and 0000927946 (variant), available at https://databases.lovd.nl/shared/individuals/00435323.

## Ethics statement

The studies involving human participants were reviewed and approved by Institutional Ethics Committees of the Faculty of Health Sciences, University of Beira Interior (Ref: CE-FCS-2013-017) Institutional Ethics Committees of the Medical Research Institute, University of Alexandria (Ref: IORG0008812). Written informed consent to participate in this study was provided by the participants’ legal guardian/next of kin.

Written informed consent was obtained from the minor(s)’ legal guardian/next of kin for the publication of any potentially identifiable images or data included in this article.

## Author contributions

CIG and JNC performed the genetic and functional studies, and wrote the first draft of the manuscript. OMO and EA diagnosed the patient and collected the clinical data. MCL conceived and supervised the study. All authors contributed to the article and approved the submitted version.
